# Application of superstatistical analysis on fluctuant surface shear in particle-laden turbulence boundary layer

**DOI:** 10.1140/epje/s10189-021-00159-x

**Published:** 2022-01-24

**Authors:** Guang Li, Wei He, Bo Yang, Hongxiang Yu, Ning Huang, Hans J. Herrmann, Jie Zhang

**Affiliations:** 1 Key Laboratory of Mechanics on Disaster and Environment in Western China, College of Civil Engineering and Mechanics, Lanzhou University, Lanzhou, 730000 China; 2 College of Atmospheric science, Lanzhou University, Lanzhou, 730000 China; 3 School of Architecture, Civil and Environmental Engineering, Swiss Federal Institute of Technology Lausanne, Lausanne, 1015 Switzerland; 4 College of Architecture and Energy Engineering, Wenzhou University of Technology, Wenzhou, 325088 China; 5 Laboratoire PMMH, ESPCI, CNRS UMR 7636, 75005 Paris, France; 6 Departamento de Física, Universidade Federal do Ceara, Fortaleza, 60020-181 Brazil

## Abstract

**Abstract:**

We report on an application of superstatistics to particle-laden turbulent flow. Four flush-mounted hot-film wall shear sensors were used to record the fluctuations of the wall shear stress in sand-laden flow. By comparing the scaling exponent in sand-free with that in sand-laden flows, we found that the sand-laden flow is more intermittent. By applying the superstatistics analysis to the friction velocity, we found that the large time scale is smaller when the flow is sand-laden. The probability density of a fluctuating energy dissipation rate measured in sand-laden flow follows a log-normal distribution with higher variances than for sand-free flow. The variance of this dissipation rate is a power law of the corresponding time scale. The prediction based on the superstatistics model is consistent with our structure function exponents $\zeta _n$ for sand-free flow. Nevertheless, it overestimates $\zeta _n$ for sand-laden flow, especially at higher Reynolds numbers.

**Graphic abstract:**

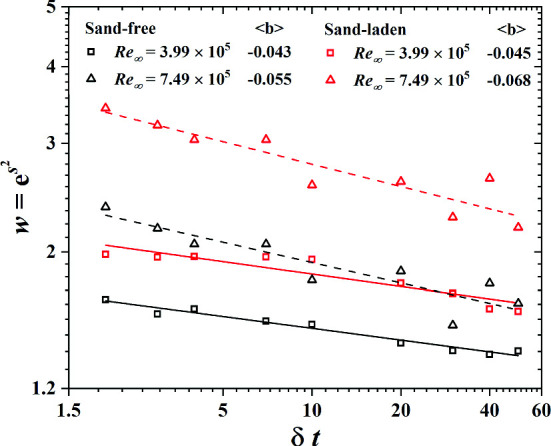

## Introduction

Horizontal wind moving in the atmospheric boundary layer (ABL) carries a large amount of horizontal momentum which could be transferred from high level downward to the bottom surface through turbulent and molecular diffusion [1]. The momentum transferred downwards finally leads to the shear force on the surface, resulting in the movement of the surface material (especially the particulate matter), causing the natural phenomena of dust storms, wind blown snow, wind erosion, sediment transport and accumulation, and many more [2]. The driving surface shear force is crucial to the study of above physical processes. In general, the mean value of surface shear force is always considered in relevant studies [2–4]. But recent studies indicate that the distribution of surface shear is also significant because of related nonlinear processes [5–7]. This motivates a more detailed investigation of the distribution of fluctuating surface shear force in the turbulent boundary layer (TBL).

The full understanding of the statistical properties of the fluctuating physical quantities in turbulent flow remains a challenging problem in theoretical physics. In recent years, there has been some experimental progress in measuring the statistics of the fluctuating parameters in both Eulerian and Lagrangian ways [8–11]. These advances together with DNS simulations [12–14] provide insight to the stochastic properties of turbulence, including the probability density of the velocity difference [15], the correlation functions [10, 16], and the Lagrangian scaling exponents [10, 16, 17]. These recent experimental results verified the early DNS results [18]. In order to provide a theoretical explanation for the most significant statistical features of turbulence, Beck [9] introduced a model based on superstatistics that can predict the measured correlation functions, the statistical dependencies between components of the velocity, and the scaling exponents in 3D. This model is able to dissociate the fast from the slow processes from a superposition of several stochastic processes. The superstatistics model specifically shows an excellent agreement at high Reynolds numbers [19, 20].

The superstatistics method has been shown to be efficient to reconstruct the statistical properties of turbulence of Newtonian fluid carrying no suspended particles. However, what would be the situation for a particle-laden flow? It is known that particles dragged by a fluid gain momentum from the fluid. There is a complex interaction between particles and fluid. For instance, large particles (1100 $\upmu \hbox {m}$ ) seem to increase the turbulent intensity near a wall, whereas small particles exhibit the opposite effect [21]. Moreover, in particle-laden flow, the intensity of turbulence of streamwise and especially vertical velocity is reduced for $z^+>10-20$ but enhanced in the very near-wall region ($z^+<5$), where “*z*” is normalized by wall unit $\nu /u_*$ [22]. Meanwhile, simulations of particle-laden flow show that particles smaller than the dissipative length scale reduce the intensity of turbulence, whereas particles somewhat larger than this length increase the intensity [23]. Similar results are reported by Lee and Lee [24], who argue that particles with Stokes numbers equal to “0.5” enhance turbulence by increasing the occurrence of quasi-streamwise vortices, whereas particles with larger Stokes numbers attenuate turbulence. These results from experiments and simulations clearly indicate that particles have an important effect on turbulent flow. It is reasonable to consider the effect of particles as an additional stochastic process. Nevertheless, there has been no application of the superstatistics model to particle-laden flow. It would be interesting to see if it can predict the statistical properties of particle-laden flow successfully.

In this study, we will introduce a superstatistical model to reproduce the statistical properties of the aeolian flow investigated in a wind tunnel experiment. Wall shear stress was recorded as an indication of turbulence, which is reasonable since the wall shear stress is the footprint of the turbulent structures in the outer region. In our experiment, we used flush-mounted hot-film wall shear sensors fabricated with a new technique to measure wall shear fluctuation in a sand-laden wall-bounded turbulent flow [25]. We present wall shear stress measurements of sand-laden flow and calculate the structure function exponents $\zeta _n$ of the friction velocity. Next, we compare our scaling exponents in sand-free and sand-laden flows to discuss the effects of airborne sand particles in the boundary layer. Finally, the superstatistics model is applied to reconstruct our experimental data and compare the prediction of $\zeta _n$ for sand-free flow and sand-laden flow. This work attempts to fill the gap between the applications of superstatistics in fluid mechanics and particle-fluid mechanics, which has significant benefits for the theoretical development of the particle-fluid flow and enhances our understanding for statistical properties of the fluctuating shear force in particle-ladenturbulent boundary layer.

## Methods

**Fig. 1:**
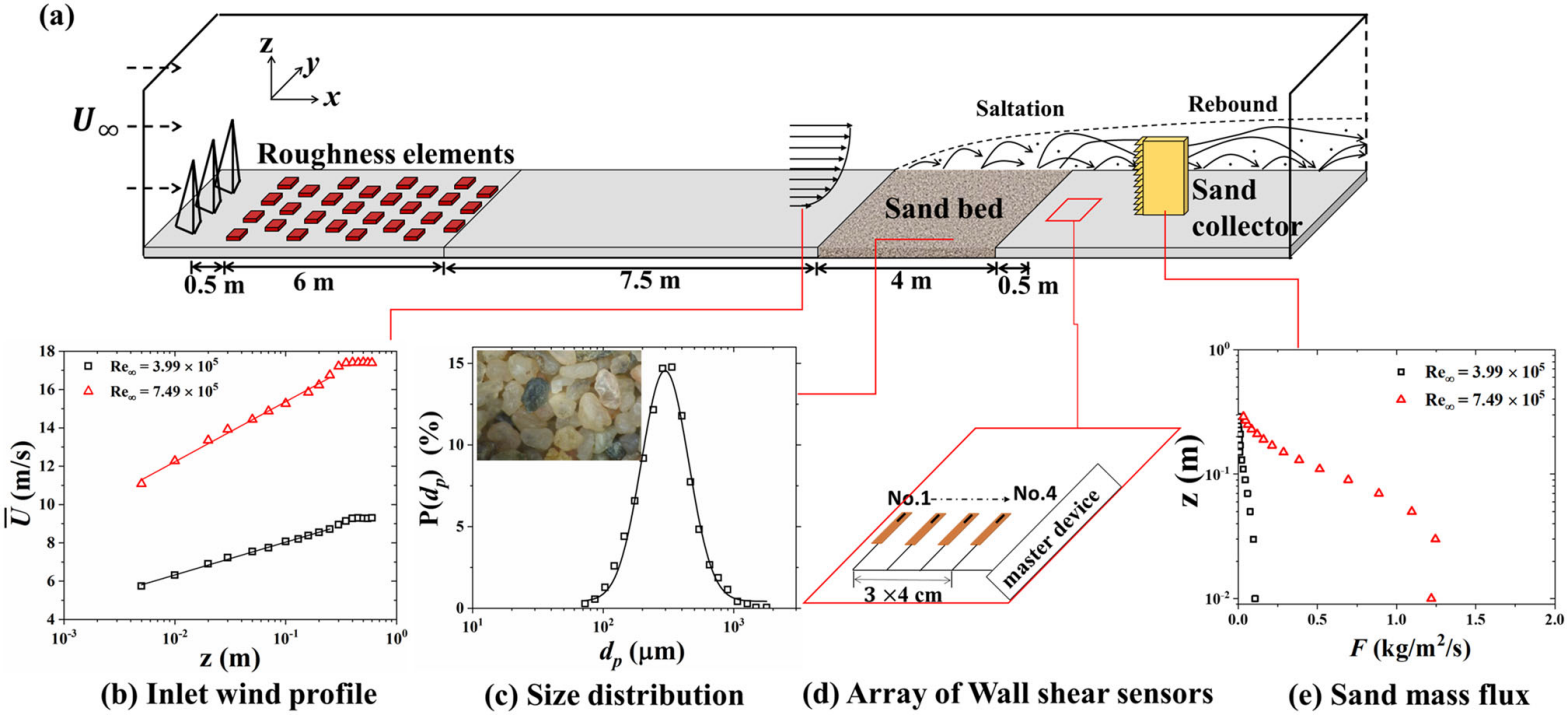
**a** Experimental setup featuring the four-point simultaneous hot-film measurements of wall shear stress with sand bed supplying airborne sand particles upstream. The wall probes of thickness 80 $\upmu \hbox {m}$ are glued on the bottom surface of the wind tunnel, the insets are **b** inlet wind profiles, **c** size distribution of sand bed, **d** the array of wall shear sensors, and **e** profiles of sand mass flux

In order to record the fluctuating wall shear stress in sand-laden flow, we use four hot-film shear sensors longitudinally glued on the bottom surface of a wind tunnel (Fig. 1d). The wind tunnel with working section $1.3\times 1.45 \times 22\,\hbox {m}^3$ provides a good tool to characterize turbulent flow in the near-wall region. In the experiment, the coordinates for the streamwise, spanwise, and vertical directions are given by *X*, *Y* and *Z*, respectively. The incoming wind velocity is adjustable between 3 and 40 m s$^{-1}$. To generate a turbulent boundary layer, we set spires and roughness elements in front of the working section. See Zhang et al. [26] for a more detailed description of the facility.

As shown in Fig. 1a, the inlet wind profiles were measured using Pitot tube anemometry. The friction velocity $u_\tau$ and roughness height $z_0$ could be obtained by fitting the measured wind speed data to the following logarithmic equation:

1 $$\begin{aligned} \overline{U}(z)=\frac{u_\tau }{\kappa }\ln {\frac{z}{ z_0}} \end{aligned}$$

where $\overline{U}$ is the time-averaged horizontal wind velocity at height *z* and $\kappa =0.41$ is the von Karman constant. For the experiments, two wind conditions are considered and the corresponding wind profiles are seen in Fig. 1b.

Following the Pitot tube anemometry, a sand bed being 4 m long and 0.03 m thick was arranged to generate a sand-laden flow. The sand is collected from the Tengger Desert in Inner Mongolia of China (inset of Fig. 1c). As seen in Fig. 1c the probability distribution of the sand particles is log-normal and the averaged diameter of sand particles is 326 $\upmu \hbox {m}$.

**Fig. 2:**
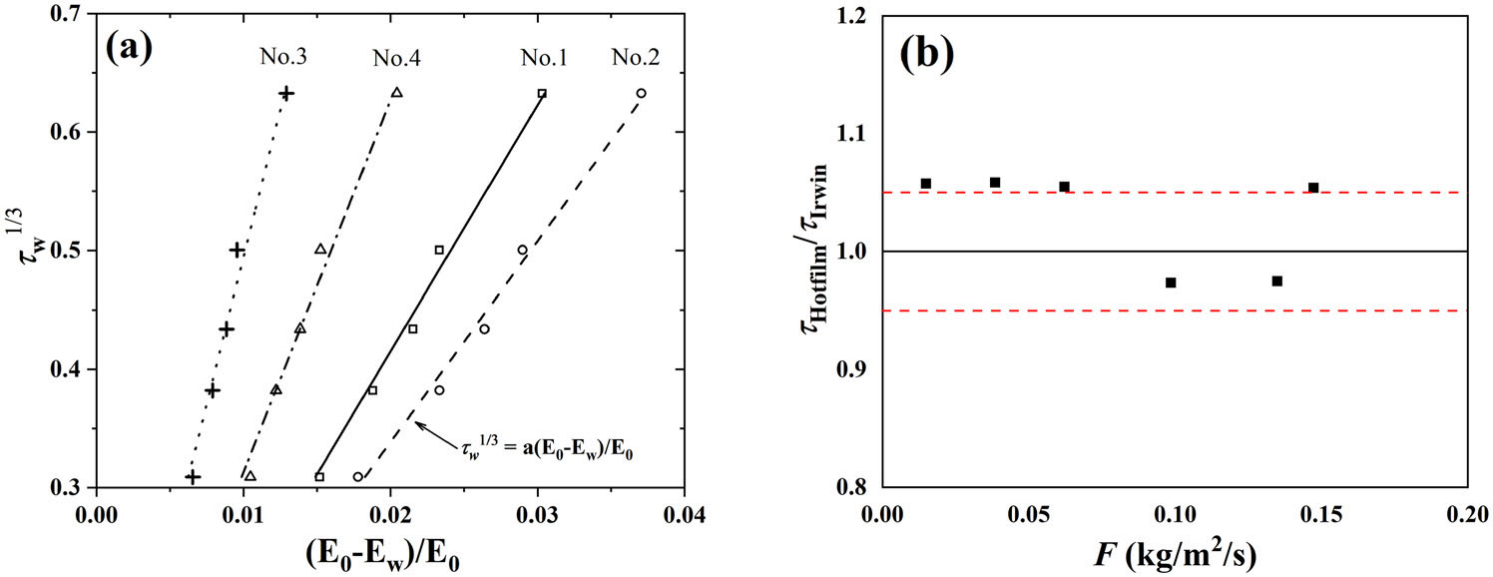
**a** Calibration of the hot-film sensors. The wall shear stress $\tau _\mathrm{w}$ is obtained by log fitting the wind profiles, $E_0$ is the voltage measured by hot-films in the absence of wind, and $E_\mathrm{w}$ is the voltage with airflow. **b** Comparison of wall shear stresses measured by hot-film and Irwin sensors under different sand mass flux

The hot-film sensors are flush-mounted behind the sand bed and a gap of 0.5 m was left between the first hot-film sensor and the sand bed in order to avoid the sensor to be buried by sand. The thickness of the hot-films is about 80 $\upmu \hbox {m}$, corresponding to $z^+=1.6$ and 2.9 at two inlet wind conditions, where $z^+=\frac{u_\tau z}{\nu }$ and $\nu$ is the kinematic viscosity of the air. The calibration and validation for the hot-film sensor are performed before the experiments. Five fan speeds of the tunnel without sand bed were select to produce clean-wind condition. Based on the measured wind profiles, five friction velocities $u_\tau$ were extracted by fitting the wind profiles with Eq. (1). These friction velocities were converted to wall shear stress via $\tau _\mathrm{w}=\rho _\mathrm{a}(u_\tau )^2$. The relationship of $\tau _\mathrm{w}$ and the normalized voltage recorded by the hot-films was subsequently established as the calibration function for the hot-film sensors (Fig. 2a). To further confirm the performance of the hot-film in sand-laden flow, we also operated some tests to compare under the same-conditions of with time-averaged shear stress between the hot film sensor and the Irwin sensor which had been successfully applied in sand-laden flow [27]. As shown in Fig. 2b, the difference between the two types of shear sensors is within 6%, indicating that hot-film sensors are reliable enough to measure aerodynamic wall shear stresses in wind–sand flow. It should be pointed out that the response speed of the hot-film sensor (2 K Hz) is much higher than that of the Irwin sensor ($\sim$ 100 Hz), so we choice the hot-film in this experiment to measure the instantaneous surface shear stress. A sand collector is installed at the end of the working section of the wind tunnel to measure the sand mass flux. The profiles of sand mass flux at two experimental conditions are shown in Fig. 1e. It is convenient to normalize the sand mass flux by:

2 $$\begin{aligned} F^+=\frac{F}{\rho _\mathrm{a} \sqrt{(s-1)g\overline{d_\mathrm{p}}}} \end{aligned}$$

where *F* is the sand mass flux, $s=\rho _\mathrm{p}/\rho _\mathrm{a}$, $\rho _\mathrm{p}$ and $\rho _\mathrm{a}$ are density of sand particle and of air, and $d_\mathrm{P}$ is the diameter of a sand particle.

**Table 1:** Parameters characterizing the experiment: sand-free flow

$U_\infty$ (m s$^{-1}$)	$\hbox {Re}_\infty$	$\hbox {Re}_\tau$	$u_\tau$ (m s$^{-1}$)	$z_0$ (mm)	$\langle \tau _\mathrm{w}\rangle$ (Pa)	$\tau _{tsd}$ (Pa)	$\eta$ (m)
9.28	$3.99\times 10^5$	$1.34\times 10^4$	0.305	0.00204	0.117	0.044	$3.70\times 10^4$
17.41	$7.49\times 10^5$	$2.45\times 10^4$	0.558	0.00127	0.388	0.151	$2.45\times 10^4$

**Table 2:** Parameters characterizing the experiment: sand-laden flow

$U_\infty$ (m s$^{-1}$)	$\hbox {Re}_\infty$	$\hbox {Re}_\tau$	$u_\tau$ (m s$^{-1}$)	$\langle \tau _\mathrm{w}\rangle$ (Pa)	$\tau _\mathrm{tsd}$ (Pa)	St	Gf	$\eta$ (m)	$\sum _{}^{}F^+$
9.28	$3.99\times 10^5$	$1.26\times 10^4$	0.287	0.108	0.056	12.5	467.4	$3.70\times 10^4$	0.155
17.41	$7.49\times 10^5$	$2.20\times 10^4$	0.502	0.337	0.187	23.4	461.4	$2.45\times 10^4$	2.256

Except the wind condition, other parameters of the experimental conditions are listed in Tables 1 and 2. The Reynolds number $\hbox {Re}_\infty =U_\infty L_0/\nu$ and $\hbox {Re}_\tau =U_\tau L_0/\nu$, where $U_\infty$ is the central wind speed measured at a height of 50 cm in the wind tunnel and the characteristic length scale $L_0=0.65$ m, which equals half the height of the wind tunnel. $\langle \tau _\mathrm{w} \rangle$ and $\tau _\mathrm{std}$ are respectively the mean and standard deviation of the surface shear stress measured by the hot-film. The Kolmogorov scale was calculated as $\eta =(\frac{\nu ^3}{\epsilon })^{\frac{1}{4}}$, where $\nu =1.48\times 10^{-5}\,\hbox {m}^2\,\hbox {s}^{-1}$ is the kinematic viscosity of the air and $\epsilon =C_\mu ^{\frac{3}{4}}\frac{k^{\frac{3}{2}}}{l}$ the global average energy dissipation. Here, $I=0.16\hbox {Re}_\infty ^{\frac{1}{8}}$ is the turbulent intensity of the incoming airflow, $k=1.5(U_\infty I)^2$ is the turbulence kinetic energy, $l=0.07L_0$ is the characteristic scale of the channel flow in the wind tunnel. The Stokes numbers (St) were estimated via $\hbox {St}=\frac{t_0 U_\infty }{L_0}$, where $t_0=\frac{\rho _p d_\mathrm{p}^2}{18\mu _g}$ is the relaxation time of particles. Here, $\rho _\mathrm{p}=2650\,\hbox {kg m}^{-3}$, $d_\mathrm{p}=3.26\times 10^{-4}$ m, and the viscosity of the air ($20\,^{\circ }\hbox {C}$) $\mu _g=1.79\times 10^{-5}\,\hbox {N m}^{-2}s$. The gravity factor (Gf) of the particles is established as $\frac{u_t}{u_\eta }$, where $u_t=\frac{gd_\mathrm{p}^2 (\rho _\mathrm{p}-\rho _\mathrm{a})}{18\mu _g}$ is the terminal velocity of sand grains, $u_\eta =(\epsilon \nu )^{\frac{1}{4}}$ is the Kolmogorov velocity scale, the air density $\rho _\mathrm{a}=1.25\,\hbox {kg m}^{-3}$ and the gravitational acceleration $g=9.8\,\hbox {m s}^{-2}$. $\sum {F^+}$ represents the integration of normalized sand mass flux in height.

## Results and discussion

**Fig. 3:**
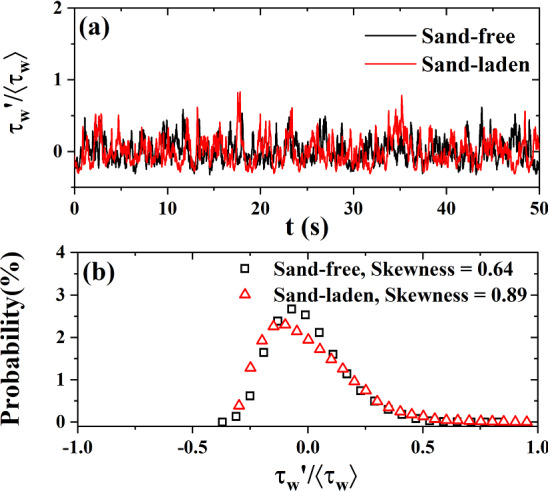
**a** Example of fluctuating wall shear stress for $\hbox {Re}_\infty =7.49\times 10^5$ in sand-free and sand-laden flows; **b** Probability density function of the normalized wall shear stress $\tau _\mathrm{w}^{'}\ /\langle \tau _\mathrm{w}\rangle$

An example of fluctuating wall shear stress measured by the hot-film is given in Fig. 3, where a stronger fluctuation in sand-laden flow can be observed. Furthermore, the probability distribution of the measured wall shear stress signal is shown in Fig. 3, with skewness of 0.64 in sand-free flow and of 0.89 in sand-laden flow. Large positive peaks seem to occur more frequently in sand-laden flow.

**Fig. 4:**
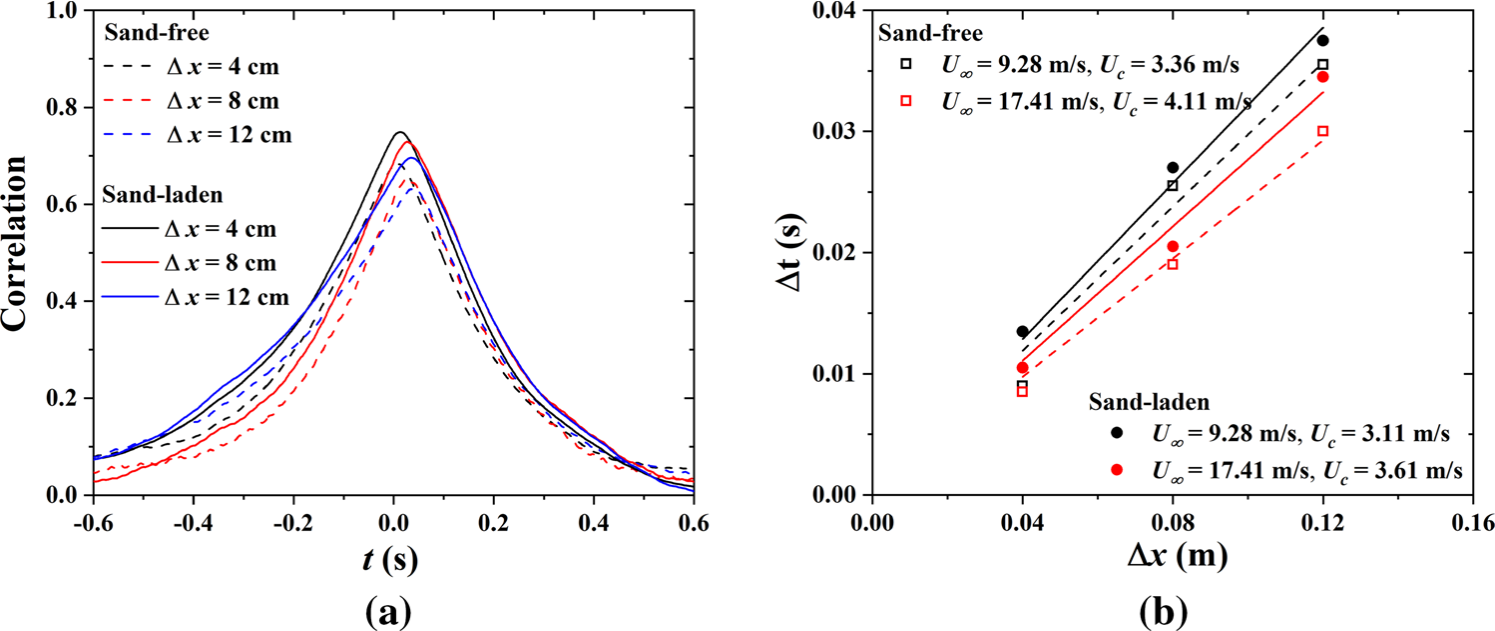
**a** Time-averaged correlation function $R_{\tau _{w,1}^{'}\tau _{w,i}^{'}}(\Delta t)$ of Eq. (3) as function of the distance $\Delta x$ between sensors, **b** convection velocity obtained by linearly fitting $\Delta t$ against $\Delta x$ in sand-free and sand-laden flows

The time-averaged correlation function between two hot-film sensors 1 and *i* ($i=2,3,4$) is defined as

3 $$\begin{aligned} R_{\tau _{w,1}^{'}\tau _{w,i}^{'}} (\Delta t)=\frac{\overline{\tau _{w,1}^{'}(t,x)\tau _{w,i}^{'}(t+\Delta t,x+\Delta x)}}{\tau _(w,1)^{'}(x)_\mathrm{rms}\tau _{w,i}^{'}(x+\Delta x)_\mathrm{rms}} \end{aligned}$$

where $\tau _{w,1}^{'}$ and $\tau _{w,i}^{'}$ are the streamwise fluctuations in aerodynamic wall shear stress, and $\Delta t$ is the time delay. Correlations were averaged over sampling periods of 5 s and normalized with the root mean square (rms) to eliminate uncertainties arising from calibration. Figure 4a illustrates strong correlations between $\tau _\mathrm{w}$ upstream and downstream. This justifies the application of Taylor’s frozen field approximation to sand-free and sand-laden flows within the range of our measurements. The convection velocity is obtained by linearly fitting the streamwise distance between hot-films sensors ($\Delta x$) against the time shift of the peak of the correlation functions, as shown in Fig. 4b.

**Fig. 5:**
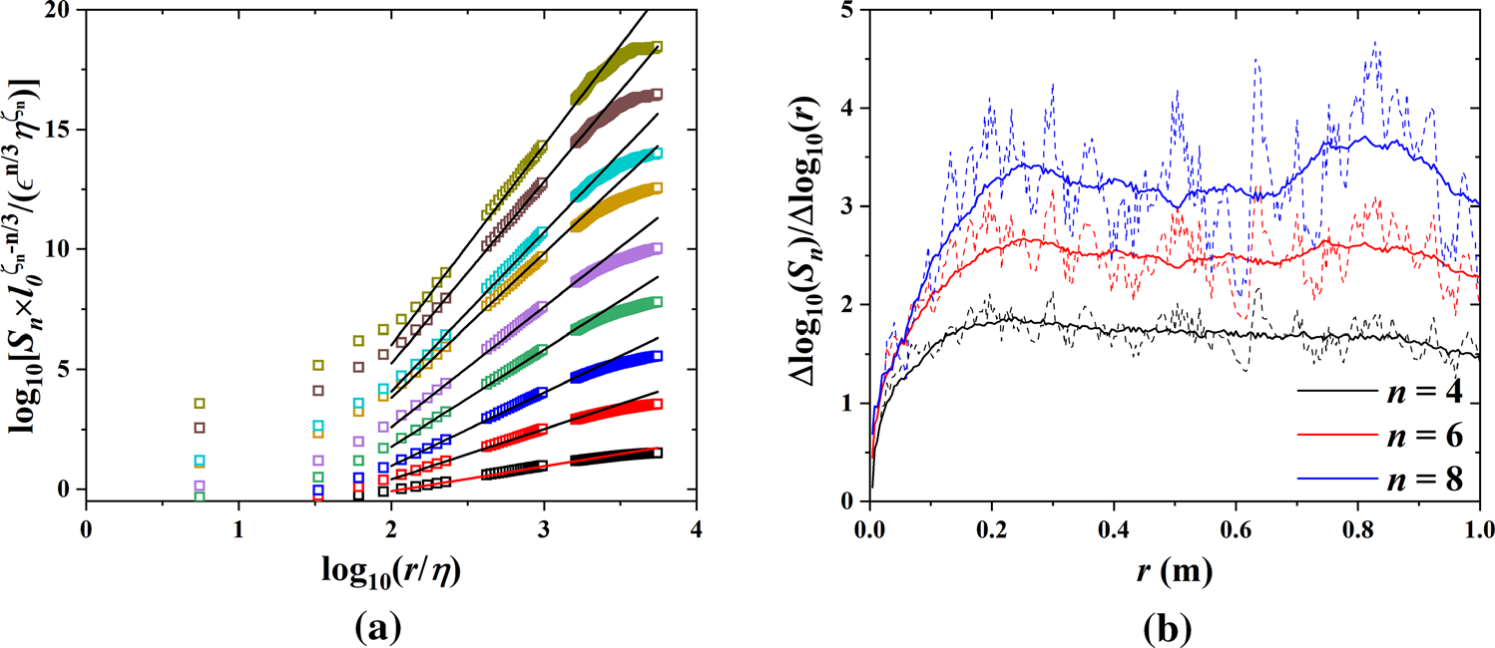
**a** Longitudinal structure functions from 1st to 9th order. Normalized by intermittent scaling relation at $U_\infty =9.28\,\hbox {m s}^{-1}$ in sand free and sand-laden flows. The solid lines stand for a linear fitting with slope $\zeta _n$ which results in $S_n=C_n(\epsilon r)^{frac{n}{3}}(\frac{r}{L_0})^{\zeta _n-frac{n}{3}}$, after cropping the structure function [removing the measurement noise (small *r*) and finite size effects (large *r*)]. **b** local slopes of $\frac{\Delta log(S_n)}{\Delta log(r)}$ versus *r* for $n = 4, 6, 8$ (dashed lines) and smoothed by a running average method (solid lines)

The structure function of turbulence is defined through the difference between friction velocities on a given length scale r as

4 $$\begin{aligned} \langle (\delta u_\tau )^n\rangle =\langle [u_\tau (x+r)-u_\tau (x)]^n\rangle \end{aligned}$$

where $u_\tau =(\tau _\mathrm{w}/\rho _\mathrm{a})^{\frac{1}{2}}$ is the friction velocity and $\tau _\mathrm{w}$ is the aerodynamic wall shear stress measured by the hot-film sensors. The time-resolved measurement of shear stress was used to compute the structure function via Taylor’s frozen hypothesis $r=U_ct$, where $U_c$ was obtained from Fig. 4b. As *n* increases, the structure functions measure more the rare events. In fully developed turbulent flow, changes in friction velocity are found to scale as a power law of r

5 $$\begin{aligned} \langle |\Delta u_\tau |^n \rangle \sim r^{\zeta _n} \end{aligned}$$

The 1st–9th order structure functions of sand-free flow and sand-laden flow are shown in Fig. 5a, where we rescaled the structure functions as $S_n \times l_{0}^{\zeta _n-n}/(\epsilon ^{n/3} \eta ^{\zeta _n})$ and the abscissa as $r/\eta$, where $\zeta _n$ is the corresponding scaling exponent of the structure function. Following the relevant work [28–32], here we used the absolute value of $\Delta u_\tau$ to reach a statistically stable result. To obtain proper spatial length scales, we cropped the structure functions by removing the segments of the data affected by measurement noise (small *r*) and by finite measurement volume (large *r*). As shown in Fig. 5b, the local slope of the normalized structure function is relatively stable within $r = 0.25$ to 0.65, this part was kept to obtain the scaling exponents. The straight lines of slope $\zeta _n$ shown in Fig. 5a establish the relation $S_n=C_n (\epsilon r)^{n/3}(r/L_0)^{\zeta _n-n/3}$, which is a more general form of the scaling relation for the structure functions, that takes into account turbulent intermittency [33].

**Fig. 6:**
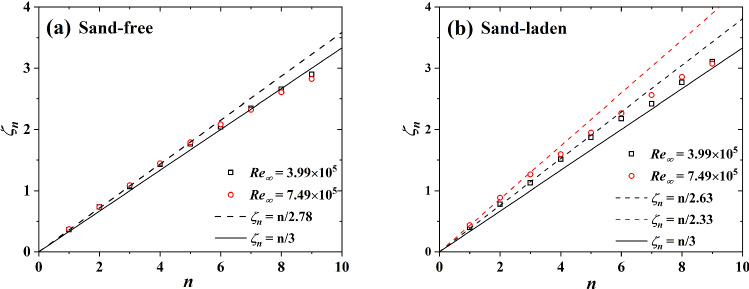
Comparison of the scaling exponents $\zeta _n$ from **a** sand-free flow and **b** sand-laden flow at $\hbox {Re}_\infty =3.99\times 10^5$ (black dots) and $\hbox {Re}_\infty =7.49\times 10^5$ (red dots). The solid lines show Kolmogorov’s theory, namely $\zeta _n=n/3$, standing for the absence of intermittency in wind velocity measurement [34]

Figure 6 shows our scaling exponents as a function of n for sand-free and sand-laden flows. In sand-free flows, the value of $\zeta _n$ seems insensitive to the Reynolds number, which is consistent with the results of [31, 33]. It was found that $\zeta _n=n/2.78$ for $n\le 5$ and $\zeta _n<n/2.78$ for $n > 5$. This behavior of the scaling exponents of the friction velocity is similar to wind velocity measurements and just differs in the slopes within the linear range. We see that the Kolmogorov’s scaling relation is not perfectly satisfied, since $\zeta _3$ is not exactly unity. In sand-laden flows, the scaling exponents are affected by the sand mass flux, $\sum _{}^{}F^+$. For $\hbox {Re}_\infty =3.99\times 10^5$, $\sum _{}^{}F^+=0.155$, $\zeta _n=n/2.63$ for $n\le 5$ and $\zeta _n<n/2.63$ for $n>5$, which is similar to what is measured for sand-free flows. For $\hbox {Re}_\infty =7.49\times 10^5$, $\sum _{}^{}F^+=2.256$, which is 14.6 times higher than for the lower Reynolds number flow. In this case we find $\zeta _n=n/2.33$ for $n\le 3$ and $\zeta _n<n/2.63$ for $n\ge 3$ which is different from the results of $\hbox {Re}_\infty =3.99\times 10^5$. Moreover, Fig. 6b illustrates stronger intermittent flow when sand-laden because the deviation between $\zeta _n$ and Kolmogorov’s law is more pronounced than in sand-free flows.

**Fig. 7:**
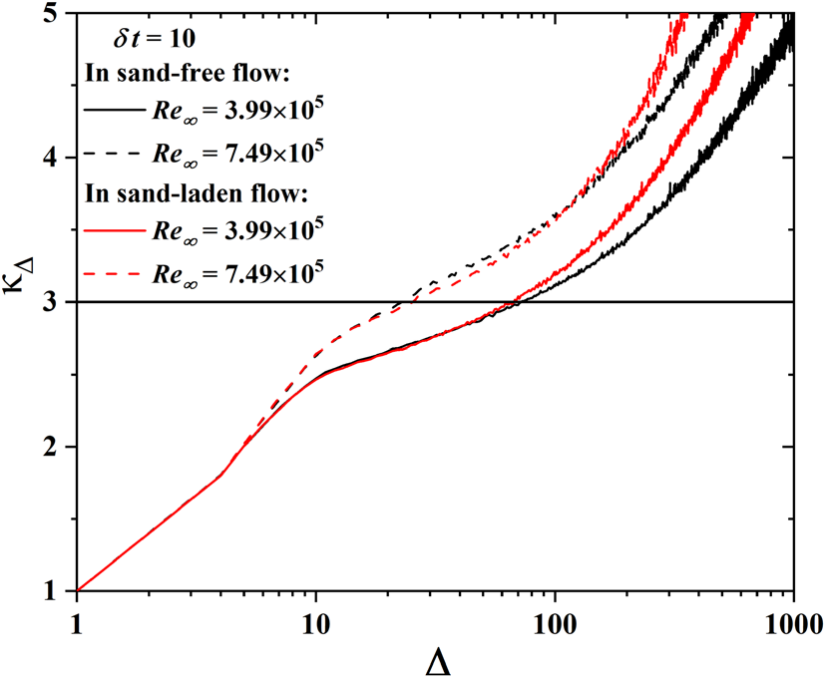
An example for the extraction of the large time scale *T* from the condition $\kappa _{\Delta }=3$ for the difference of friction velocities $\delta u_\tau$ on a given time scale $\delta t$=10. We show results for sand-free (black lines) and sand-laden flow (red lines) at $\hbox {Re}_\infty =3.99\times 10^5$ (solid lines) and $\hbox {Re}_\infty =7.49\times 10^5$ (dashed lines)

Beck [9] proposed a superstatistical model to analyze stochastic processes including turbulence. This model describes a superposition of several stochastic processes including a fast one given by the basic stochastic differential equation (SDE) and a slow one for the parameters of the SDE. To apply the superstatistical SDE to wall shear fluctuations, we extend this superstatistical model to the wall shear stress by replacing u by $\Delta u_\tau$. First, a linear approach is considered to define the velocity difference:

6 $$\begin{aligned} \Delta \dot{u}_\tau = -\gamma F(\Delta u_\tau ) + \hat{\sigma }L(t) \end{aligned}$$

here the damping constant $\gamma$ describes the dissipation of turbulent energy, $F(\Delta u_\tau )$ is a drifting force, $\hat{\sigma }$ describes the strength of the noise, and *L*(*t*) is a Gaussian white noise. In a most natural way, $\beta =\gamma /\hat{\sigma }^2$ acts as a simple function describing the fluctuating energy dissipation [35]. $\beta$ varies from cell to cell on the large spatio-temporal scale *T*. To further specify the stochastic process $\beta _{T,l}(t)$, the large time scale *T* is needed. As introduced by Straeten and Beck [36], the total time series of $\Delta u_\tau$ is divided into N equal slices of length $\Delta$. A function $\kappa _\Delta$ is introduced as s

7 $$\begin{aligned} \kappa _\delta =\frac{1}{N}\sum \nolimits _{l=1}^N\kappa _{\Delta ,l},\;\; \hbox {with}\;\; \kappa _{\Delta ,l}=\frac{\langle (\Delta u_\tau )^{4}\rangle _{\Delta ,l}}{{\langle (\Delta u_\tau )^{2}\rangle }^2_{\Delta ,l}} \end{aligned}$$

where $\kappa _{\Delta ,l}$ is the kurtosis of the *l*th time slice. The superstatistical large time scale *T* is then defined by the condition $\kappa _\Delta =3$. Figure 7 shows an example for the extraction of the large time scale *T* from the time series of the difference of friction velocities $u_\tau$ on a given time scale $\delta t=10$. With increasing Reynolds number, this large time scale decreases. For sand-laden flow, *T* is smaller, indicating a less stable $\beta$ due to the disturbance of the saltating sand particles.

**Fig. 8:**
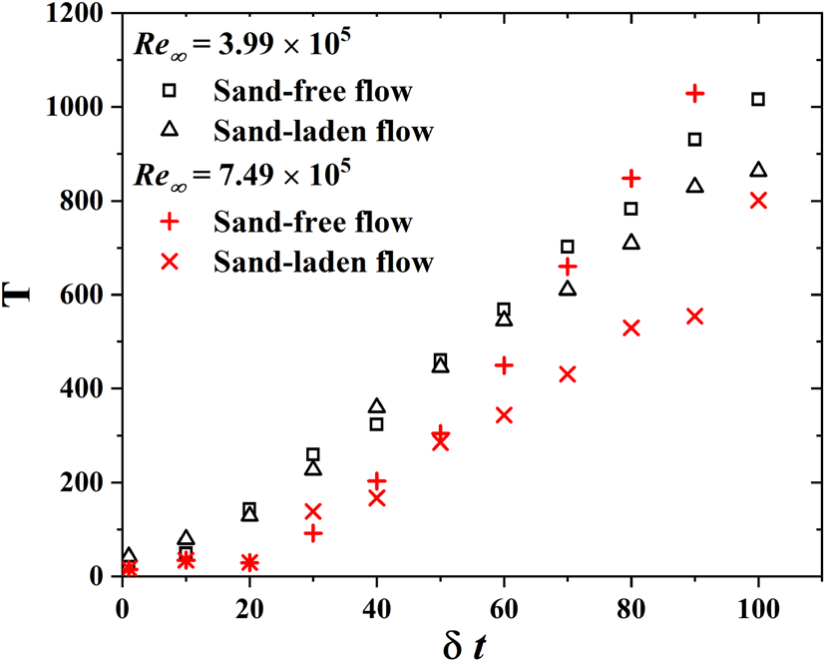
The superstatistical large time scale *T* as a function of the time scale $\delta t$ for sand-free and sand-laden flow at $\hbox {Re}_\infty =3.99\times 10^5$ and $\hbox {Re}_\infty =7.49\times 10^5$

Figure 8 shows that *T* is roughly proportional to the time scale $\delta t$ that is used to calculate the difference of the friction velocities. In sand-free flow, the large time scale *T* exceeds the one for sand-laden flow and this trend tends to be more pronounced as $\delta t$ increases. In addition, this trend is enhanced at the larger Reynolds number of $\hbox {Re}_\infty =7.49\times 10^5$ and we speculate that this might be due to a higher mass flux of saltating sand particles.

**Fig. 9:**
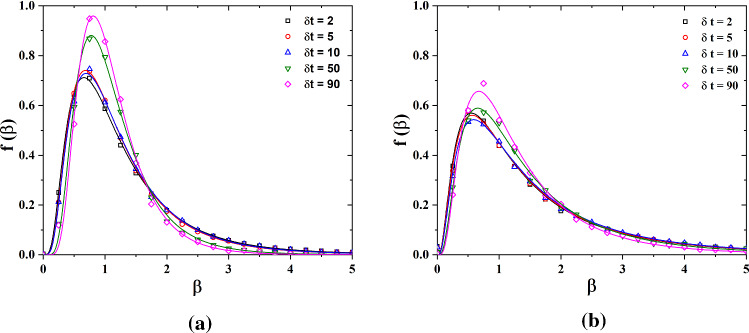
The distribution of the stochastic process $\beta$ for **a** sand-free and **b** sand laden flow at $\hbox {Re}_\infty =3.99\times 10^5$. The solid lines are fits using the distribution of Eq. (8)

After the large time scale *T* is determined, welta $u_\tau$ process as $\beta _{T,l} (t)=\langle \beta e(\Delta u_\tau )^2 \rangle \beta _{T,l}$. The probability density function f($\beta$) is obtained through the histogram of the time series of $\beta (t)$. Figure 9 shows the distribution of $\beta$(t) for various $\delta t$ for sand-free and sand-laden flow. Motivated by the cascade picture of turbulence and previous successful models [19, 37, 38], the stochastic process $\beta (t)$ is assumed to be close to a log-normal distribution for sand free flow:

8 $$\begin{aligned} f(\beta )=\frac{1}{\beta s\sqrt{2\pi }}\exp \left[ -\frac{\log (\beta /m)^2}{2s^2}\right] \end{aligned}$$

here *m* and *s* stand for mean and variance. For sand-laden flow, the prediction of Eq. (8) is also consistent with experimental results. Since the early papers by Kolmogorov in 1962, there is consensus that the probability density of energy dissipation $\epsilon$ is approximately log-normal in turbulent flow. For sand-free and sand-laden flow, the log-normal distribution of $\beta (t)$ implies a simple power-law relation between $\beta$ and $\epsilon$.

When the stochastic processes reach a local equilibrium after a time *T*, the local distribution of $\Delta u_\tau$ can be approximated by a Gaussian distribution:

9 $$\begin{aligned} P(\Delta _\tau | \beta )=\sqrt{\beta /2\pi }e^{-\frac{1}{2}\beta (\Delta u_\tau )^{2}} \end{aligned}$$

This Gaussian distribution will vary since $\beta$ fluctuates on large time scales. By substituting Eq. (8) into this distribution, we get a superposition of Gaussians with variance parameter $\beta ^{-1}$:

10 $$\begin{aligned} P(\Delta u_\tau )=1/\sqrt{2\pi }\int _{0}^{+\infty }\beta ^{\frac{1}{2}}f(\beta )e^{-\frac{1}{2}\beta (\Delta u_\tau )^2 } \hbox {d}\beta \end{aligned}$$

**Fig. 10:**
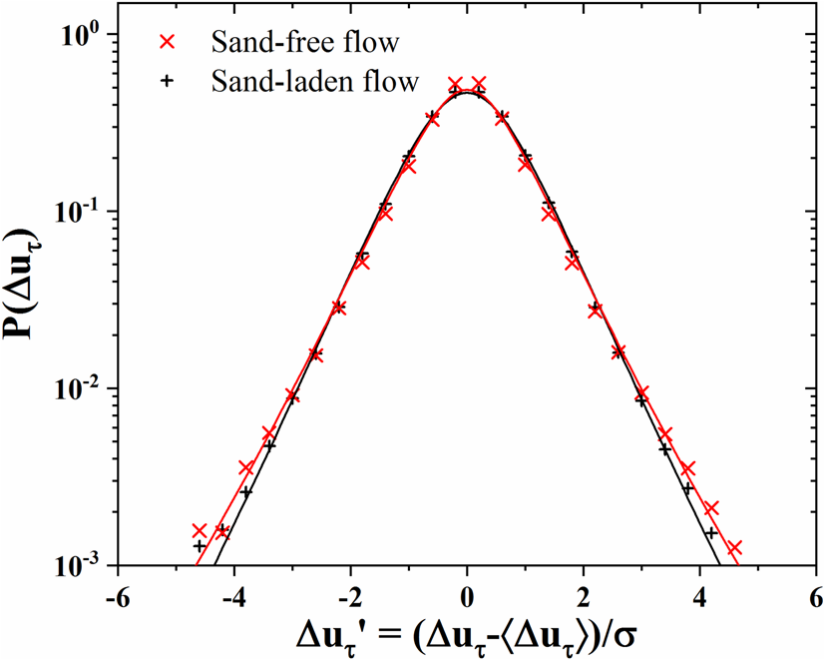
Experimentally measured probability distribution $P(\Delta u_\tau )$ at the shortest time scales for $\delta t=0.5$ ms for sand-free and sand-laden flow. The experimental data is fitted by Eqs. (8) and (10) with $s^2=0.47$ and 0.68, respectively

This formula is in good agreement with the experimentally measured histogram of $\Delta _\tau$, as shown in Fig. 10.

**Fig. 11:**
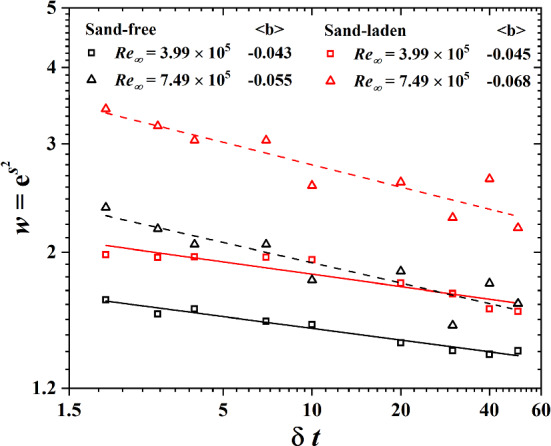
Power law between *w* and $\delta t$ of No. 2 wall shear sensor for sand-free and sand-laden flow at $\hbox {Re}_\infty =3.99\times 10^5$ and $\hbox {Re}_\infty =7.49\times 10^5$. At right we show the mean value of four sensors $\langle b\rangle$, which is introduced into Eqs. (12) and (13) to obtain $\zeta _n$

According to Beck [9], the superstatistical model can predict the structure function exponent of the velocity difference. He obtained the moments as

11 $$\begin{aligned} \langle (\delta u_\tau )^n\rangle =(j-1)!!m^{-\frac{n}{2}}w^{\frac{1}{8}n^2} \end{aligned}$$

with $w=e^{s^2}$ Here *m* and *s* are the mean and variance in Eq. (8) where $m~(\delta t)^a$ and $w~(\delta t)^b$. The notation (j-1)!! stands for a product of all odd positive integers up to $j-1$. Figure 11 shows the power law, $w~(\delta t)^b$ for sand-free and sand-laden flows. For sand-free flow, this scaling law is in excellent agreement with our measured data points. For sand-laden flow, however, the correlation between fitting curves and experimental data is reduced by the saltating sand particles. Figure 11 also presents the averaged result of four hot-film sensors. With increasing Reynolds number, the value $\langle b\rangle$ increases, especially for sand-free flow.

From Eq. (11), we can imply

12 $$\begin{aligned} \zeta _n=-\frac{n}{2}a+\frac{n^2}{8}b \end{aligned}$$

**Fig. 12:**
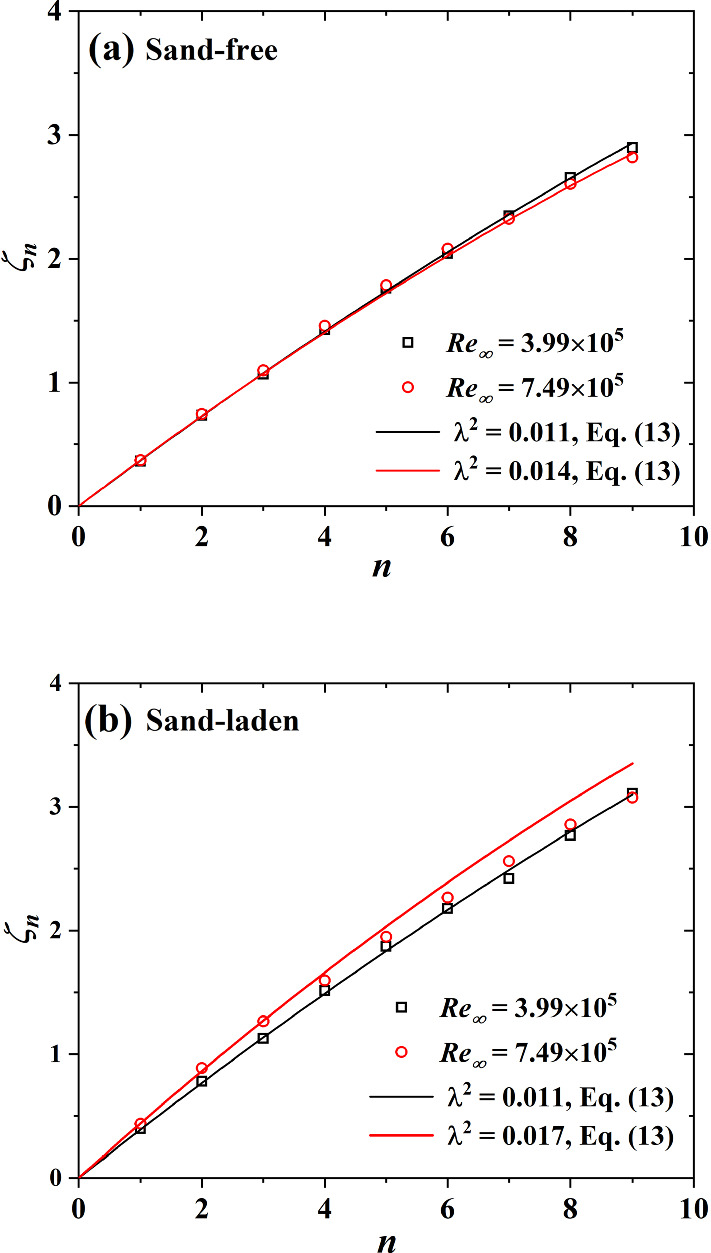
Structure function exponent $\zeta _n$ as measured in our experiment and as predicted by the superstatistical model of Eq. (13)

It is usually assumed that the scaling exponent $\zeta _n$ equals to unity for wind velocity measurements [28], however, this is not applicable to friction velocity measurements due to the different slopes as shown in Fig. 6. In sand-free flows, from $\zeta _{2.78}=1$ we get $a=\frac{2.78}{4}b-\frac{2}{2.78}$ and thus:

13 $$\begin{aligned} \zeta _n=\left( \frac{1}{2.78}+\frac{2.78}{2}\lambda ^2 \right) n-\frac{1}{2}\lambda ^2n^2 \end{aligned}$$

where we defined a positive parameter $\lambda ^2=-0.25b$ [10]. In sand-laden flows, Eq. 13 corresponds to the slope of the dashed line shown in Fig. 6. As shown in Fig. 11, b is obtained by a power law fit between w and $\delta t$. As indicated in Fig. 12, Eq. (13) is in good agreement with the data for sand-free flow. However, this formula overestimates $\zeta _n$ for sand-laden flow, especially for higher Reynolds number due to the enhanced sand mass flux.

The turbulence structure in sand-laden flow is important to understand the interaction of transported particles and turbulent atmosphere boundary flow, which is still unknown so far. Our work is a preliminary attempt to find a theoretical analysis on it and could be helpful to improve the numerical model of aeolian transport, especially in intermittent aeolian transport flow [39]. Aeolian processes are common in solar system, such as Mars or the Comet Churyumov–Gerasimenko [40]. However, the viscous sublayer is larger on these extra-terrestrial worlds due to the low gravity. In that situation, the turbulent structure created by particle motions should be more obviously since the air flow is laminar flow, which could give us a clearer image about the turbulent flow of aeolian particles. Thus, it could be also useful to explain the formation of aeolian landforms on Earth and these extra-terrestrial worlds, such as dunes, ripples, and mega-ripples [41–43], through more nature analysis of the particle motions.

## Conclusion

In this paper, we obtained the scaling exponents $\zeta _n$ of structure function of turbulence, which is defined through the difference between friction velocities $(\Delta u_\tau )^n$ on a given length scale r, by using hot-film wall shear sensors in sand-free and sand-laden flows. The results show that Kolmogorov’s scaling relation for the structure function is only to a limited extent applicable to both flows. In sand-free flow, the scaling exponent is insensitive to the Reynolds number. Scaling exponents for sand-laden flow exhibit a more intermittent flow condition. In order to analyze the statistics of turbulence from a different point of view, we introduced the superstatistics model to analyze friction velocity data from sand-laden flow. The large time scale *T* is proportional to the time scale $\delta t$ that is used to calculate the difference of the friction velocities. The large time scale *T* is shorter for sand-laden flow and this trend becomes more pronounced as $\delta t$ increases. In addition, this trend increases at higher Reynolds number due to an enhanced sand mass flux. We found that the probability distribution of the stochastic process $\beta (t)$ in sand-laden flow is close to a log-normal distribution with larger variance than in the sand-free case. This feature implies that $\beta$ is a simple power-law function of energy dissipation. We verified the power law, $w\sim \delta t^b$ in sand-free flow, finding that this scaling law is in excellent agreement with our measured data points. In the sand-laden flow, however, the correlation between the fitting curves and experimental data is smaller. At the end, we found that the superstatistics theory predicts the scaling exponents $\zeta _n$ in sand-free flow very well. However, it overestimates $\zeta _n$ in sand-laden flow, especially in the context of higher Reynolds numbers due to the enhanced sand mass flux.

## Data Availability

This manuscript has associated data in a data repository. [Authors’ comment: The datasets generated during the current study are available from the corresponding author on reasonable request.]
